# Impact of West Virginia Abortion Ban on West Virginia Physician Trainee Practice Intentions

**DOI:** 10.13023/jah.0704.07

**Published:** 2025-12-01

**Authors:** Rebecca Wald, Kristy Ward, Sarah Dotson

**Affiliations:** West Virginia University; West Virginia University; West Virginia University

**Keywords:** Abortion, Appalachia, Dobbs, Roe v Wade, trainees

## Abstract

In June 2022, the Dobbs v Jackson Women’s Health Organization overturned the constitutional right to abortion established by Roe vs. Wade. Shortly after, West Virginia (WV) began enforcing a near total ban on abortion. Statewide restrictions on access to reproductive health care have important implications for the recruitment and retention of physicians. This project aimed to determine overall awareness of the 2022 changes in abortion laws among physician trainees, and whether the law influenced the intention of resident/fellow physicians to practice medicine in the state following completion of their medical training. A total of 178 resident and fellow physicians at West Virginia University were recruited via an email invitation. Survey responses demonstrated that 91.6% of trainees were aware of the change in abortion legislation. Among all respondents, 52.2% reported being less likely to practice in WV because of the ban. Compared to residents, fellows were much more likely not to want to practice in WV (82.7% vs. 48.2%, pp =0.015). Open ended commentary revealed that this is an important issue for physician trainees. These findings have important implications for recruitment and retention of physicians to abortion-restricted states and may also suggest changes to where medical students are applying for residency.

## INTRODUCTION

In the 1973 decision *Roe v Wade*, the United States (U.S.) Supreme Court recognized the right of a woman to decide whether to continue or terminate a pregnancy until fetal viability (at or around 24 weeks gestation). This federal ruling overturned state laws that criminalized abortion. In June 2022, *Dobbs v Jackson Women’s Health Organization* reversed this decision, ruling that there is no individual Constitutional right to abortion. Subsequently, West Virginia (WV) was the second state to enact a near total ban on abortion on September 13, 2022. That law prohibits abortion at all stages of pregnancy except in cases of a “non-medically viable fetus,” ectopic pregnancy, or medical emergency, as well as in limited cases involving rape or incest (requiring a police report or medical evaluation of injuries). Interpretation of these exceptions is left to the “reasonable medical judgement of a licensed medical professional.” Penalties for violating the law include loss of licensure for medical providers and felony charges of up to 10 years in prison for non-licensed individuals.

Whereas restrictive abortion legislation directly impacts the care of women seeking abortion services, there also has been considerable discussion about recruitment, retention, and professional burden among healthcare providers in states with abortion bans. Providers practicing in Obstetrics and Gynecology are obviously directly affected in the way they deliver care to patients, but other specialties including Diagnostic Radiology and Orthopedic Surgery are seeing impacts in their residency recrutment.^1^–^3^ Psychiatrists report increased emotional burden in providing care to patients seeking abortion, especially when placed in the position of determining whether an abortion is “medically necessary” and an expected increased need for psychiatric services in these states.^4^ Family Medicine providers have reported changes in their counseling practices, more challenging clinical decision-making, worry of legal risks, and lack of trust in their patients’ self-reporting of reproductive medical history since the *Dobbs* decision.^5^

WV is home to 100 ACGME specialty and subspecialty training programs, over half of which are at West Virginia University (WVU), the largest academic medical center in the state. The purpose of the current study was to explore awareness of the WV abortion legislation among residents and fellows at WVU, and its effect on their future intentions to practice medicine in WV following completion of their medical training.

## METHODS

An email invitation to participate in this survey was sent via listserv to all active WVU Graduate Medical Education trainees (both residents and fellows) on two occasions in January and April 2024. The sample was chosen after multiple attempts to contact graduate medical education offices at other institutions across the state. Participants were given an overview of the study purpose and provided consent to participate. Participation was voluntary, and no incentives were offered.

Data was collected and managed using REDCap (Research Electronic Data Capture), a secure, web-based software platform designed to support data capture for research studies. Three novel survey questions, developed specifically for this project shortly after the announcement of the 2022 WV abortion legislation addressed 1) awareness of the ban, 2) whether respondents would choose residency/fellowship training in WV if they could apply again, and 3) whether that legislative change influenced the respondent’s intent to practice in the state upon completion of training. Responses were on a 5-point Likert scale. Self-reported demographic data included age, gender, training program, post-graduate training level and previous education in WV. Open-ended comments regarding personal reactions to the state abortion ban were invited at the end of the questionnaire. A complete copy of the survey tool is included in Appendix A.

To evaluate the factors associated with the decision to practice in WV after the state’s abortion law was passed, Likert variables were performed on a 5-point scale and were analyzed as continuous variables with independent t-test analysis. Specific variables studied included high school attendance in WV, college attendance in WV, medical school attendance in WV, primary care training programs versus specialty medical programs, postgraduate year (PGY1) level versus other PGY level, resident versus fellow, and gender (male versus female). Question 2 regarding whether respondents would still choose their training program in WV after awareness of the abortion ban was excluded from final analysis because response options were ultimately felt to be ambiguous.

## RESULTS

A total of 178 of 598 (29.7%) trainees responded to the survey. Table 1 summarizes the demographic characteristics of the study population. Respondents were more likely to be female (51.5%) and age 25–34 (83.5%). Prior personal associations with the state of WV included: 22.7% attended high school; 20.9% attended an undergraduate institution; and 31.9% attended medical school in WV. 82.3% of respondents were in residency and 17.7% were in fellowship. Responses were evenly split among those having current or past residency training in a primary care specialty (49.4%) compared to a non-primary-care specialty (50.6%).

With respect to level of awareness of the September 2022 legislation restricting abortion in the state, 91.6% reported being very aware or somewhat aware of the ban. With respect to future practice intentions, 52.2% of respondents reported being somewhat or much less likely to practice in WV because of the abortion ban, 30.4% reported the legislation had no influence on their future practice intentions, and 17.4% reported being somewhat or much more likely to practice in the state.

Results were further analyzed based on demographic variables including trainee type (resident v fellow), post-graduate year, specialty type (primary care v non-primary care), attendance of high school in WV, attendance of undergraduate school in WV, attendance of medical school in WV, and gender (Table 2). When compared to residents, fellows were much more likely to report an intention to leave WV because of the abortion ban (82.7% vs. 48.2%, mean 4.41+/−0.78 vs. 3.39+/−1.26), *p* <0.001). No fellows who responded to the survey reported being more likely to practice in WV because of the abortion ban. First-year residents (who chose WV for training *after* the 2022 abortion legislation change) reported being *more* likely to practice in WV compared to trainees who started prior to the abortion ban (28.9% v 11.3%, mean 3.16+/−1.37 vs. 3.72+/−1.19, *p* =0.015). Female trainees reported being much less likely to stay to practice medicine in WV because of the abortion legislation compared to male trainees (71.4% v 38.5%, 3.98+/−1.10 vs. 3.32+/−1.12, *p* <0.001). Responses based on attending high school, undergraduate, or medical school in WV were not significantly different, and there was no difference between primary care and other specialties.

A linear regression was performed with likelihood to practice in WV as the dependent variable (Table 3). Post-graduate year, medical school in WV, high school in WV, and age were removed from the final model with a significance > 0.6. The final model is shown in Table 3. Overall, awareness of the ban, ACGME program, and undergraduate in WV were not statistically significant. As the ban impact increases, the likelihood of practicing in WV decreases (B −0.39, *p* <0.001). Gender has a negative impact on practicing in WV (other gender and female less likely B −0.29 *p* <0.001)). Fellows were less likely to practice in WV (B 0.60, *p* = 0.004).

Twenty-five open-ended comments were given by respondents. Overall, 17 comments had a “pro-choice” connotation, 3 portrayed “pro-life” sentiments, and 5 were neutral. A selection of open-ended comments that relate directly to the decision of whether to practice in WV after the abortion ban are shown below.

“Do not want to provide Emergency care in a state that doesn’t value patient rights and endangers their everyday lives.” – Other (Emergency Medicine), PGY-2

“I am glad to fall back on state law to prevent me from being forced to do something I find objectionable.” – Family Medicine, PGY-2

“This does not impact my training or future practice, so it is a non-issue.” – Other surgical specialty, PGY-3

“Although my personal beliefs on this topic do not align with the current anti-abortion laws, which I consider anti-access to health care and anti-West Virginians, as an IM physician it does not impact my day-to-day practice in WV.” – Internal Medicine, PGY-3

“In my field of practice, the abortion laws don’t affect my ability to care for patients’ psychiatric conditions. However, these laws do affect mental health of patients, which affects my work in that way. However, if I worked in OBGYN this would definitely affect my decision to continue practicing in WV.” – Psychiatry, PGY-2

“This topic has had a huge impact on my desire to stay in West Virginia after completing residency. It breaks my heart as a West Virginian, as a woman, and as a medical provider to see that we are sliding backwards. Not only does this affect me personally, but I have already seen the effects of the ban on some of my patients.” – Psychiatry, PGY-2

## DISCUSSION

Overall, graduate medical trainees at WVU were highly aware of post-*Dobbs* changes to state abortion laws, suggesting that this generation of physicians is paying attention to how politics is influencing the practice of medicine. Notably, over half of respondents reported being somewhat or much less likely to practice in WV after completion of medical training because of the ban. This may have a significant future impact on recruitment and retention of young physicians to a rural state that already suffers shortages of healthcare providers. Furthermore, the issue appears to be even more important for physicians in subspecialty fellowship training programs, with negative implications for the future availability of subspecialists in the state. Interestingly, PGY-1 respondents were more likely than others to want to stay and work in WV despite the abortion ban. This represents the only group who ranked and matched in their WV-based programs *after* the WV abortion ban was enacted. Thus, this cohort made a deliberate decision to choose a residency program in a state that severely restricts abortion. This suggests that medical students who believe it is important to train and work in a state with access to abortion may be self-selecting not to train in a state with significant abortion bans. This could mean a future shift towards a physician workforce that is less supportive of women’s reproductive rights within states with restrictions on abortion access.

Other studies investigating the impact of the *Dobbs* decision on physician practice intentions have shown similar findings. Decreases in applications to OBGYN residency programs nationwide were noted from 2022 – 2023.^6^ Seventy percent of students surveyed in Indiana reported they would be less likely to apply to residency in abortion-restricted states and more than 50% reported a decreased likelihood of choosing OBGYN as a specialty.^7^ At the University of Utah, 7% of graduating residents across all specialties changed their practice location after the *Dobbs* decision, and many wanted to advocate for safe, legal abortion.^8^ Qualitative commentary from the study revealed personal concerns from physicians in accessing reproductive health care for themselves or their partners.^8^ Similarly, 17.6% of residents graduating from an OB/GYN program that included abortion training indicated that the *Dobbs* decision changed the location of intended future practice or fellowship plans, and that graduates who had planned to practice in now abortion-restricted states were eight times more likely to change practice location compared to those who intended to practice in an abortion-protected state prior to *Dobbs*.^9^ Lastly, a 2023 analysis by the AAMC Research and Action Institute found that fewer new graduates of US medical schools applied to residency programs in states that banned or restricted access to abortion than to residency programs in states where abortion remained legal.^10^ Students and practicing physicians from across the country cite both patient care concerns and personal factors as contributing to their decisions about future training and practice.^11^

The current study has several limitations. Participation was voluntary, and therefore the sample may be biased towards individuals who have strong opinions on the topic of abortion. Overall, the sample is felt to be largely representative of the GME population at WVU, with participation from trainees across specialties and years. Female respondents were slightly overrepresented at 51.5% compared to 38% at the institution, which may demonstrate selection bias. The subgroup analysis of trainees in fellowship is limited by the low number of respondents (n=29). Using self-reported future intention as a proxy for behavior limits the impact of the findings, and further research is needed to demonstrate how physician workforce changes play out in the years following the *Dobbs* decision. Study findings may not be generalizable to all abortion-restricted states as this issue is complex and influenced by local geography, culture, political climate and medical training environments.

## CONCLUSION

Findings from this study have important implications for recruitment of medical students to residency programs in abortion-restrictive states, as well as recruitment and retention of physicians after completion of training. When compared to prior literature focusing on OBGYN and primary care specialties, the current study highlights that subspecialists in training (29 fellows in non-OBGYN specialties) report being less likely to stay and practice in WV because of the restrictive abortion legislation. Current physicians in training demonstrate an astute awareness of current politics related to reproductive health rights, and many feel politics will influence their decision of where to practice medicine long term. Legal protections and support of advocacy training for trainees are important for shaping future healthcare policy. Further research is needed to explore the changing demographics of residents self-selecting to train in states based on availability of abortion care and how that might polarize the future medical workforce based on state laws.

## Supplementary Information



## Figures and Tables

**Table 1:** Demographic Characteristic of Survey Respondents

Demographics	n (%)
**Gender**	
Male	65 (39.9%)
Female	84 (51.5%)
Non-binary	1 (0.6%)
Other	13 (8.0%)
**Age**	
25–29	62 (37.8%)
30–34	75 (45.7%)
35–39	23 (14.0%)
≥40	4 (2.4%)
**Prior education in WV**	
High school in WV	37 (22.7%)
Undergraduate in WV	34 (20.9%)
Medical School in WV	52 (31.9%)
**Post-graduate training year (PGY)**	
PGY-1	38 (23.5%)
PGY-2	32 (19.8%)
PGY-3	39 (24.1%)
PGY-4	28 (17.7%)
PGY-5	12 (7.4%)
PGY-6	9 (5.6%)
PGY-7	3 (1.9%)
PGY-8	1 (0.6%)
**Program Type**	
Primary Care	80 (49.4%)
Internal Medicine	53 (32.7%)
Family Medicine	12 (7.4%)
Obstetrics & Gynecology	6 (3.7%)
Pediatrics	3 (1.9%)
Med-Peds	6 (3.7%)
Non-Primary Care	82 (50.6%)
Anesthesia	6 (3.7%)
Psychiatry	12 (7.4%)
Neurology	5 (3.1%)
Radiology/Radiation Oncology	7 (4.3%)
General Surgery	3 (1.9%
Other surgical specialty	17 (10.5%)
Other	32 (19.8%)
**ACGME training type**	
Resident	135 (82.3%)
Fellow	29 (17.7%)

**Table 2:** Likelihood to Practice in West Virginia – Sub-Group Analysis

Demographic Factors (N)		More Likely to Practice in WV N(%)	Neutral N (%)	Less Likely to Practice in WV N (%)	Mean +/− SD	*p-*value
**Trainee Type (164)**						
Resident	135	26 (19.3)	44 (32.6)	65 (48.2)	3.39 +/− 1.26	<0.001
Fellow	29	0 (0)	5 (17.2)	24 (82.7)	4.41 +/−0.78	
**Post-Graduate Year (162)**						
PGY-1	38	11 (28.9)	9 (23.7)	18 (47.4)	3.16 +/− 1.37	0.015
>PGY-1	124	14 (11.3)	40 (32.3)	70 (56.5)	3.72 +/− 1.19	
**Training Program (162)**						
Primary Care*	80	13 (16.3)	25 (31.3)	42 (52.5)	3.51+/−1.19	NS
Specialty Care	82	12 (14.6)	24 (29.3)	46 (56.1)	3.66+/−1.30	0.457
**High School (163)**						
West Virginia	37	6 (16.2)	12 (32.4)	19 (51.4)	3.41+/−1.17	NS
Other	126	20 (15.9)	37 (29.4)	69 (54.8)	3.62+/−1.28	0.365
**Undergraduate (163)**						
West Virginia	34	19 (14.7)	38 (29.5)	72(55.8)	3.26+/−1.16	NS
Other	129	7 (20.6)	11(32.4)	16 (47.1)	3.65+/−1.27	0.111
**Medical School (163)**						
West Virginia	52	19 (17.1)	30 (27.0)	62 (55.9)	3.52+/−1.15	NS
Other	111	7 (13.5)	19 (36.5)	26 (50.0)	3.59+/−1.31	0.722
**Gender** ** **(149)**						
Female	84	11 (16.9)	29 (44.6)	25(38.5)	3.98 +/− 1.10	<0.001
Male	65	6(7.1)	18 (12.4)	60 (71.4)	3.32 +/− 1.12	

NOTES:

* Family Medicine, Internal Medicine, Pediatrics, Med-Peds and OBGYN

** Other gender categories were excluded from analysis given low n

**Table 3:** Linear Regression Predicting Practice in West Virginia

Predictor	B (Unstandardized)	SE (B)	β (Standardized)	t	*p*-value
Constant	4.650	0.389	—	11.95	<0.001
Awareness	−0.076	0.102	−0.046	−0.75	0.458
Ban impact	−0.388	0.054	−0.448	−7.11	<0.001
Resident/Fellow	0.602	0.205	0.186	2.93	0.004
ACGME Program	0.016	0.018	0.059	0.91	0.364
Undergraduate in WV	−0.333	0.199	−0.109	−1.67	0.097
Gender	−0.290	0.048	−0.378	−6.01	<0.001
