# Supplementary material for: Impact of West Virginia Abortion Ban on West Virginia Physician Trainee Practice Intentions
Source: J Appalach Health. 2025 Dec 1;7(4):116–27. doi: 10.13023/jah.0704.07 (PMC13239641; doi:10.13023/jah.0704.07)
Supplement: Supplementary file 1 [file 7.4.7_Wald_Additionalfile.docx]

In the 1973 decision *Roe v. Wade*, the US Supreme Court recognized that the right to liberty in the constitution protected personal privacy and included the right to decide whether to continue a pregnancy and protected a woman’s federal right to seek abortion services. In June 2022, the Supreme Court decision in *Dobbs v. Jackson Women’s Health Organization* overturned the *Roe v. Wade* decision, ruling there was no constitutional right to abortion.

On September 13, 2022, West Virginia began enforcing a near total ban on abortion, which prohibits abortion at all stages of pregnancy, except in the case of “non-medically viable fetus”, ectopic pregnancy, or medical emergency, as well as in limited cases involving rape or incest.

This information comes directly from Supreme Court of the United States and The Supreme Court of Appeals of WV.

Questions:

Prior to participating in this this survey, were you aware of the above information?

- Very aware
- Somewhat aware, but I was unclear of all the details
- Not at all aware

To what extent would the WV abortion ban impact your decision to obtain your residency or fellowship training in WV?

- Highly likely
- Somewhat likely
- No influence
- Somewhat unlikely
- Very unlikely

To what extent would the WV abortion ban influence your decision to practice medicine in WV following completion of your medical training?

- Much more likely to practice in WV
- Somewhat more likely to practice in WV
- No influence
- Somewhat less likely to practice in WV
- Much less likely to practice in WV

Demographics:

Are you a resident or fellow?

- Resident
- Fellow

What ACGME training program are you in or did you complete?

- Internal Medicine
- Family Medicine
- OB/Gyn
- Pediatrics
- Medicine/Pediatrics
- Psychiatry
- Neurology
- Radiology or Radiation Oncology
- General Surgery
- Other Surgical specialty
- Other

What is your current post-graduate year in training?

- PGY1
- PGY2
- PGY3
- PGY4
- PGY5
- PGY6
- PGY7
- PGY8
- PGY9
- PGY10+

Did you attend undergrad at WVU?

- Yes
- No

Did you attend medical school at WVU?

- Yes
- No

Did you attend high school in WV?

- Yes
- No

What is your age?

- 20-25
- 23-30
- 30-35
- 35-40
- 40-45
- 45-50
- 50+

What is your gender identity?

- Female
- Male
- Trans female
- Trans male
- Gender neutral
- Non-binary
- Other

Comments on this topic?

(open ended response – optional)
